# Supplementary material for: Bisphenol A Exposure Interferes with Reproductive Hormones and Decreases Sperm Counts: A Systematic Review and Meta-Analysis of Epidemiological Studies
Source: Toxics. 2024 Apr 17;12(4):294. doi: 10.3390/toxics12040294 (PMC11054375; doi:10.3390/toxics12040294)
Supplement: Supplementary file 1 [file toxics-12-00294-s001.zip › toxics-2949068-supplementary.pdf]

Table S1. Search strategy.

Search target	Specific terms
Bisphenol A	"Bisphenol A" OR "Bisphenol-A" OR "BPA" OR "2,2-Bis (4-hydroxyphenyl) propane" OR "4,4'-Isopropylidenediphenol"
Male reproduction	"Male reproduction" OR "Male subfertility" OR "Male infertility" OR "Subfertile men" OR "Infertile men" OR "Asthenozoospermia" OR "Oligospermia" OR "Azoospermia" OR "Asthenospermia" OR "Teratospermia" OR "Oligoasthenoteratozoospermia"
Sperm parameters	"Sperm parameter(s)" OR "Sperm" OR "Semen" OR "Spermatozoa" OR "Sperm concentration" OR "Total sperm count" OR "Sperm motility" OR "Sperm vitality" OR "Spermatozoa viability" OR "Sperm morphology" OR "Sperm volume" OR "Ejaculation volume"
Reproductive hormones	"Reproductive hormone(s)" OR "Estradiol" OR "E_2" OR "free androgen index" OR "FAI" OR "Follicle stimulating hormone" OR "FSH" OR "Free testosterone" OR "fT" OR "Inhibin B" OR "INB" OR "Luteinizing hormone" OR "LH" OR "Sex hormone binding globulin" OR "SHBG" OR "Testosterone" OR "Total testosterone" OR "T"

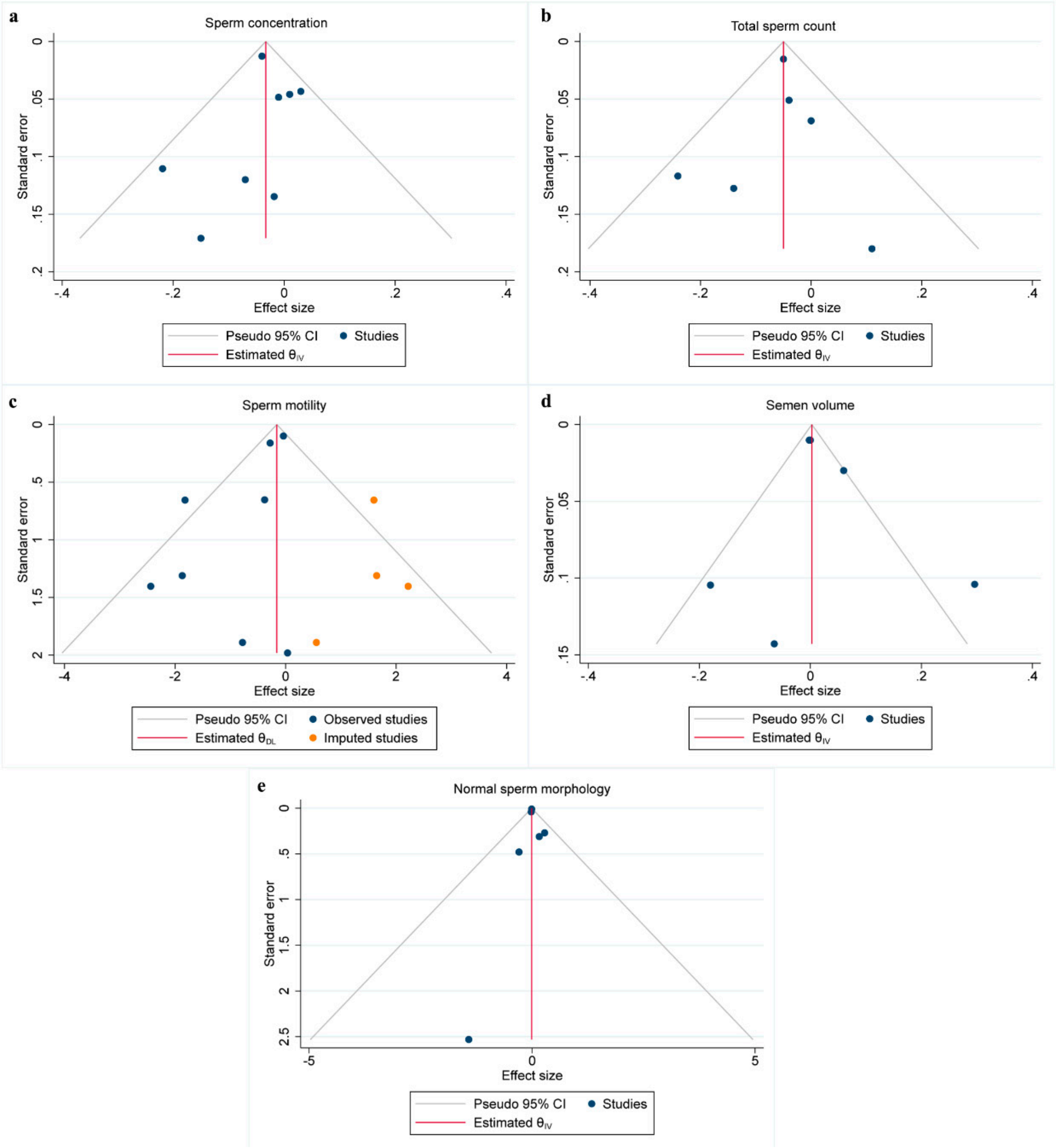

Figure S1. Funnel plots for the analysis of the correlation of urine BPA levels and sperm concentration (a), total sperm count (b), sperm motility (c), semen volume (d) and normal sperm morphology (e).
